# The Financial Divide in Congenital Heart Surgery: Global Challenges and Solutions

**DOI:** 10.31083/RCM43778

**Published:** 2025-07-31

**Authors:** Sneha Prothasis, Janett Francis, Priya Prothasis, Vivienne Mathews, Jeevan Francis

**Affiliations:** ^1^Department of Cardiothoracic Surgery, Aberdeen Royal Infirmary, AB25 2ZN Aberdeen, UK

## 1. Introduction

Congenital heart disease (CHD) is the most common congenital defect worldwide. In high-income countries (HICs), advances in care mean that a majority of children with CHD now survive into adulthood. In stark contrast, outcomes in low- and middle-income countries (LMICs) remain poor resulting in approximately 220,000 deaths annually, the vast majority occurring in LMICs [1].

While HICs benefit from specialised networks of heart centers with reliable healthcare funding, LMICs often face critical shortages of pediatric cardiac surgeons, anaesthetists, perfusionists, and intensivists [2]. In many LMICs, healthcare financing is largely dependent on out-of-pocket expenditures. This potentially exposes families to catastrophic financial risks when seeking surgical care for their children [3]. Insurance policies, if available, often fail to cover the full cost of surgery, particularly for complex congenital conditions requiring prolonged hospitalization and repeat interventions [4].

Beyond immediate health consequences, the societal burden of untreated CHD is also significant. Children who survive without corrective surgery may experience long-term disabilities, impairing their development, education, and further limiting their future economic productivity. At a macroeconomic level, the loss of human capital from preventable deaths and disabilities places a long-term strain on already stretched healthcare systems in LMICs.

Whilst charitable surgical missions and local program developments have made a positive impact in specific regions, these initiatives are often episodic and cannot meet the overwhelming need. In this editorial, we explore five critical economic themes that perpetuate the financial divide in congenital heart surgery, examining their implications and highlighting opportunities for global solutions.

## 2. Out-of-Pocket Costs and Catastrophic Health Expenditure

In many LMICs, healthcare financing is dominated by out-of-pocket payments. For example, in India, 62.6% of total health expenditure is paid out-of-pocket [5]. For each operated child, parents in India lose an average of two weeks salary, which can be a substantial cost for households who are already only living pay-check to pay-check [6]. This means that for many families, these costs often exceed their annual household income. To afford these procedures, families often resort to mortgaging assets or taking out high-interest loans, increasing their financial strain [6].

In countries such as Nigeria, the cost of open-heart surgery can be 2–3× the per capita gross national income (GNI) [7]. This forces families to deplete savings, sell property, or forgo treatment. In LMICs without access to local surgical services, the cost burden is exacerbated. In these situations, governments may send patients to HIC’s for lifesaving surgeries which can account for up to 10–12% of the national healthcare budget [8].

## 3. Limited Insurance and Inadequate Public Funding

Public health insurance programs in LMICs often exclude high-cost interventions like congenital cardiac surgery. For instance, India’s Ayushman Bharat program provides a cover of approximately 6000 USD per family each year. However, complex congenital surgeries often require multiple interventions and prolonged intensive care unit (ICU) stays, making this inadequate [9].

In Kenya, the National Hospital Insurance Fund covers certain operations but do not routinely fund pediatric cardiac surgery [10]. As a result, most children requiring surgery must either depend on philanthropic organizations or go untreated.

In LMICs, children and families without health insurance consistently report being unable to access specialized care [11]. Insurance coverage can also be assessed as a predictor of outcomes, as infant survival is significantly lower in infants with inadequate, underinsured, or with no insurance. Infants within this group were found to have a 30% higher risk of mortality during the post-neonatal period than privately insured infants [12].

## 4. Cost of Infrastructure and Sustainability Challenges

A pediatric cardiac surgical unit requires investment in operating rooms, catheterization laboratories, pediatric ICUs, and a trained multidisciplinary workforce [13]. Compared to HICs, LMICs allocate a smaller percentage of their GDP to healthcare, limiting their ability to fund essential services [14].

A global census reported that on average, in LMICs, there are only 0.13 pediatric cardiac surgeons per million in the under 15s population [15]. In many LMICs, hospitals rely on intermittent surgical missions rather than sustainable local programs. While these missions address immediate needs, they result in inconsistent services.

Many studies propose government funding as a solution to improving healthcare outcomes. However, the result of this may not always improve specialized care clinics; this is seen in India where the Kerala state government set up many financial initiatives [16]. Hospitals that took part in the initiative were left unable to clear costs that incurred as a result of reimbursement rates, leaving the specialized clinics inadequately staffed and stripped important revenue from the hospital which could have been better used in other areas [16].

## 5. Impact on Access and Equity

Disparity in access to CHD care exists within LMICs, but even within these countries, disparities in resources and access is seen in different geographical areas. In minority and underdeveloped neighborhoods, clinics and facilities are either non-existent or sparsely spread [17]. Facilities tend to congregate within the larger metropolitan cities. However, a large proportion of those living within rural areas are unable to access these facilities [18]. Studies show that children from rural areas are significantly less likely to obtain timely diagnosis and surgical care compared to urban patients [19]. In addition, these children present for surgery almost three times above the median age compared to their urban counterparts [18].

Transport costs, accommodation during hospitalization, and loss of income for caregivers add indirect costs, often doubling the overall economic burden [18]. In Ethiopia, where there are only a few centers offering cardiac surgery, families often travel hundreds of kilometres, leading to delayed interventions and worse outcomes [10].

## 6. Broader Societal Costs

Beyond individual families, untreated CHD imposes significant societal costs particularly in LMICs. Children who survive without corrective surgery often suffer from chronic heart failure, growth retardation, and neurodevelopmental delays, reducing future economic productivity. In LMICs, 66% of preventable deaths and 58% of disability-adjusted-life-years (DALYs) are due to CHD [2]. Investing in congenital cardiac services has allowed some LMICs to save millions of dollars and has the potential to yield substantial long-term economic benefits [8].

For every child that undergoes surgery for CHD in LMICs, it is reported that an average of two weeks of job days and over a month worth of man-days are lost [6]. Many families report incurring debt relating to their child’s health expenses which contribute to about 75% of their total debt [6]. In a study based in Columbia, it was estimated that each caregiver of a child undergoing CHD surgery incurred a cost of $1303, with an average of 140 days taken for leave from jobs. The families most affected by this were those earning less than the normal living wage per month [20].

## 7. Conclusion

The financial divide in congenital heart surgery reflects broader inequities in global child health. Without significant investment in health financing mechanisms, infrastructure development, and workforce training, millions of children will continue to face preventable morbidity and mortality. Strategies should include prioritizing emergency surgical hubs in high-mortality regions, followed by systematic workforce training, supported by cost-benefit data from LMICs that have successfully developed similar initiatives. Bridging this divide demands urgent, coordinated action by governments, donors, and the global health community to ensure that no child’s life depends solely on their ability to pay.
